# Preoperative Cone Beam Computed Topography Assessment of Maxillary Sinus Variations in Dental Implant Patients

**DOI:** 10.3390/diagnostics14171929

**Published:** 2024-09-01

**Authors:** Alexandru Misăiloaie, Ionuț Tărăboanță, Cristian Constantin Budacu, Anca Sava

**Affiliations:** Faculty of Dental Medicine, “Grigore T. Popa” University of Medicine and Pharmacy from Iasi, 16 Universitatii Str., 700115 Iasi, Romania

**Keywords:** cone beam, maxillary sinus, anatomical variations

## Abstract

This study aimed to evaluate the pathological factors and anatomical variations in the maxillary sinus in patients undergoing dental implant treatment using cone beam computed tomography (CBCT). CBCT, as a key imaging technique in dentistry, offers high-resolution images to assess bone morphology and quality, crucial for preoperative dental implant planning. Material and methods: The study included a cohort of 200 patients recommended for CBCT as part of their preoperative evaluation. The methodology involved detailed CBCT image analysis to identify and document various anatomical variations due to pneumatization, exostosis, hypoplasia, polyps, cysts, foreign bodies, and anthroliths within the maxillary sinus. Results: Pneumatization was the most common variation, present in 77.5% of subjects. Polypoid lesions were found in 17.5% of patients, with a higher prevalence in younger age groups (57.1% in ages 20–35). Cysts and polyps affected 17.5% of subjects, predominantly males (65.7%). Anthroliths were observed in a minimal percentage (2%), and foreign bodies were found in 1.5% of the patients. Positive correlations were observed between the patient’s age and both mucosal thickness and polypoid lesions and between the patient’s gender and bone thickening (*p*-values < 0.05). Conclusions: The study concluded that CBCT is essential in the preoperative assessment of the maxillary sinus in dental implant candidates due to its superior imaging capabilities, allowing for the identification of critical anatomical variations and pathologies. This thorough evaluation is imperative to ensure the success of implant placement and to mitigate potential complications. However, further research with larger, more diverse populations is recommended to confirm these findings.

## 1. Introduction

Cone beam computed tomography (CBCT) is a paraclinical imaging technique with wide application in dentistry. It is based on a conical or pyramidal beam capable of recording a variety of projections in a single rotation [1,2]. This method allows the localization and visualization of contrasting oral structures, such as teeth, bones, or air cavities [2]. CBCT analysis makes it possible to assess bone morphology and quality based on high-resolution images and is useful in the preoperative planning of dental implants. In terms of radiation, CBCT units typically deliver very low doses [1,2].

Despite its many recognized advantages, the CBCT technique has a variety of drawbacks, such as the presence of artifacts, a limited volume, or the inability to evaluate soft tissues [3]. For these reasons, some authors recommend the use of CBCT in addition to other paraclinical imaging methods. The anatomical elements of the oral region can be evaluated by panoramic radiographs, which benefit from low cost and low radiation dose, but have the disadvantage of overlapping anatomical structures and the inability for cross-sectioning the image [4].

Along with the qualitative assessment of bone structures, paranasal sinuses, such as maxillary, frontal, ethmoid, and sphenoid sinuses, can also be localized and analyzed by the CBCT technique. The analysis is useful in identifying maxillary sinus anatomical variations, such as pneumatization, exostosis, or hypoplasia [1,5,6], considering that maxillary sinuses are of major significance in dental practice [1]. The literature shows that, in terms of visualization of paranasal sinuses, the CBCT technique is considered to be the gold standard due to its low radiation level, low procedure time, and the quality of the obtained image [4].

Treatment with dental implants is safe, popular, and more frequently accessed by patients. The technique may present difficulties, particularly when implants are placed in the posterior regions of the maxilla, where ridge height and bone density may be low. Various sinus floor elevation techniques have been developed to overcome these shortcomings [7]. Performing this therapy involves a number of risks and possible complications that may compromise the result; therefore, a detailed knowledge of the anatomy of the entire region and of the potential variations in the sinus is necessary. Although the technique of creating the base with grafts is well documented, studies are needed to evaluate the local arterial supply through the supero-posterior alveolar, infraorbital, descending palatine, and sphenopalatine arteries and their anatomic variations. The arteries are responsible for healing and osseointegration, and a good knowledge of their morphology and anastomoses is vital for achieving favorable results and avoiding possible complications [8,9]. The maxillary sinus is the most extensive of the paranasal sinuses, located at the level of the maxillary bone. It consists of an airy, pyramid-shaped cavity lined by a mucous membrane called the Schneiderian membrane. The maxillary sinus is connected to the nasal cavity through a small opening called the ostium. However, the maxillary sinus anatomy can be influenced by factors such as the patient’s dental status, age, or gender [10].

The sinus floor elevation procedure can be performed by both a lateral fenestration or transcrestal approach [11]. When the access is lateral, the maxillary sinus must be accessed but with a risk of injuring the supero-posterior alveolar artery. Hemorrhage following injury of the artery is directly proportional to the diameter of the vessel; therefore, preoperative CBCT imaging is required to determine its size [12]. The literature shows that one of the most common complications occurring in sinus floor elevation therapy is the perforation of the Schneiderian membrane, and in order to avoid this, a detailed analysis of the nasal septal defects, as well as the thickness and configuration of the maxillary sinus walls, is required [7,13].

The loss of teeth in the posterior maxillary region leads to several morphologic changes in the maxillary sinuses, such as the phenomenon known as pneumatization. This anatomical variation is characterized by a change in the dimensions of the antral cavity and a change in the location of the main vascular elements, hence the importance of preoperative imaging assessment in ensuring clinical success [9]. At the same time, the location of the supero-posterior alveolar artery varies depending on factors such as age, gender, presence of teeth on the arches, or sinus dimensions [9,14]. 

The aim of this study was to evaluate the anatomic variations in the maxillary sinuses observed on CBCT images in patients proposed for dental implant treatment of different ages and genders. The null hypothesis stated that no correlation could be made between age or gender and the maxillary sinus anatomic variations.

## 2. Materials and Methods

### 2.1. Study Design

The study was conducted in accordance with the provisions of the Declaration of Helsinki and in compliance with the rules imposed by the Research Ethics Committee of the “Grigore T. Popa” University of Medicine and Pharmacy of Iasi. (Approval no. 186/17 May 2022).

This cross-sectional study was performed on a total sample of 200 subjects aged between 20 and 81 years old. Their CBCT scans were analyzed in a private dental radiology clinic in Iasi, Romania.

All the participants approved their participation in the study by signing an informed consent. In order to be included in the study, subjects had to meet certain criteria, such as the need for a radiographic examination using CBCT at the maxillary level; age over 18 years; cooperative patients who agreed to participate in the study and signed an informed consent; painful, inflammatory, or infectious symptoms starting from a tooth in the posterior region of the maxillary arch; and patients with a recommendation for implant treatment at the maxillary level in the posterior region. Exclusion criteria were the inability to completely and clearly visualize the targeted anatomical structures on the CBCT image, subjects younger than 18 years old, or the patient’s unwillingness to participate in the study or to sign the informed consent. According to age, subjects were included in 3 age groups: group A: 20–35 (*n* = 68); group B: 36–60 (*n* = 115); group C: 61–81 (*n* = 17).

The radiological examination was performed using an ORTHOPANTOMOGRAPH OP 3D PRO cone-beam computed tomography (Kavo Dental, Biberach, Biberach, Germany) with operating conditions of a 6 cm field of view, 0.2 mm voxel, and a scan time of 40 s.

### 2.2. Image Analysis

To analyze the images, software compatible with the CBCT device, OnDemand 3D Project Viewer, v.1, module 3D (Cybermed Company, Daejeon, Republic of Korea), was used.

In the evaluation of anatomic variations, we aimed to follow the antral septum, hypoplasia, pneumatization, and exostoses. The presence of lesions was also assessed; sinonasal mucosal thickening with values greater or lower than 3 mm and bone thickening were measured using a digital ruler. The presence of polypous formations, cysts, malignant-looking formations, foreign bodies, antrolith, or sinus opacification phenomena was also followed. All these elements were identified and analyzed by two observers, and Cohen’s Kappa statistical test was used to determine the level of acceptance between them. The resulting K-values were interpreted according to a classification developed by Landis and Koch: values <0.00 = poor; 0.00–0.20 = slight; 0.21–0.40 = fair; 0.41–0.60 = moderate; 0.61–0.80 = substantial; 0.81–1.00 = almost perfect [15].

### 2.3. Statistical Analysis

The Statistical Package for the Social Sciences software version 29.0.0 (SPSS Inc., Chicago, IL, USA) was used for the statistical analysis of the data. For all the evaluated parameters, in addition to Cohen’s Kappa analysis, we checked the normality of distribution using the Kolmogorov–Smirnov test.

Initially, descriptive statistics were performed on the data, calculating the frequency of occurrence of each maxillary anatomic variation or lesion. A Pearson’s statistical correlation test was used to correlate the patient’s age and sex with the analyzed variables.

## 3. Results

In the present study, the CBCT examinations of 200 subjects with a mean age of 48.81 ± 14.84 years, variance of 219.83, median of 47.0, and mean rank of 63.0 were analyzed. Of the subjects included in the study, 113 (56.5%) were male and 87 (43.5%) were female.

Following the analysis of CBCT images (Table 1) we could observe that in 163 subjects (81.5%) pneumatization was present at the alveolar level in 53 (32.52%) cases and in multiple sites in 110 (67.48%) patients. Antral septum was found in 97 subjects (48.5%). None of these variables could be correlated with the patient’s gender or age. Hypoplasia was found in 13 (6.5%) of the patients. Exostoses were found in three subjects, representing 1.5% of the total analyzed subjects.

In 119 (59.5%) subjects, a thickening of the mucosa of more than 3 mm was observed on CBCT images, and in the remaining 81 (40.5%) of them, the mucosa had a thickness of less than 3 mm. According to the results indicated by the Chi-square test, the phenomenon of mucosal thickening was correlated with the age group of the patient, considering the Pearson test value of 65.605 with two degrees of freedom and a significance of <0.001. Thus, in patients in the age group of 20–35 years, ≤3 mm of mucosal thickening was identified in 98.5% of the subjects. In the age group of 61–81 years, ≤3 mm mucosal thickening was observed in 88.8% of subjects.

**Figure 1:**
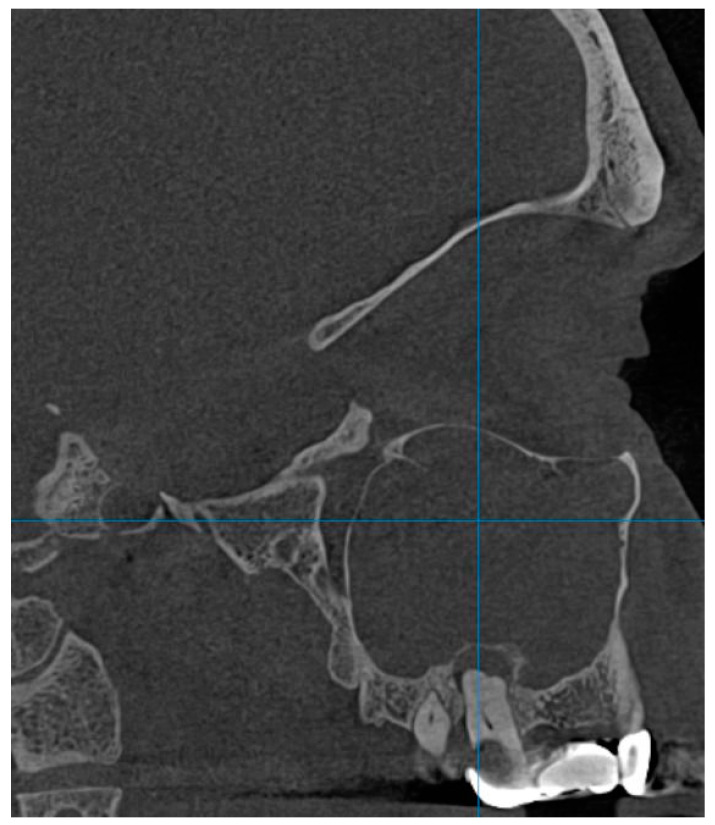
Completely opacified left maxillary sinus following chronic maxillary sinusitis consecutive to chronic periapical osteitis 2.7.

**Figure 2:**
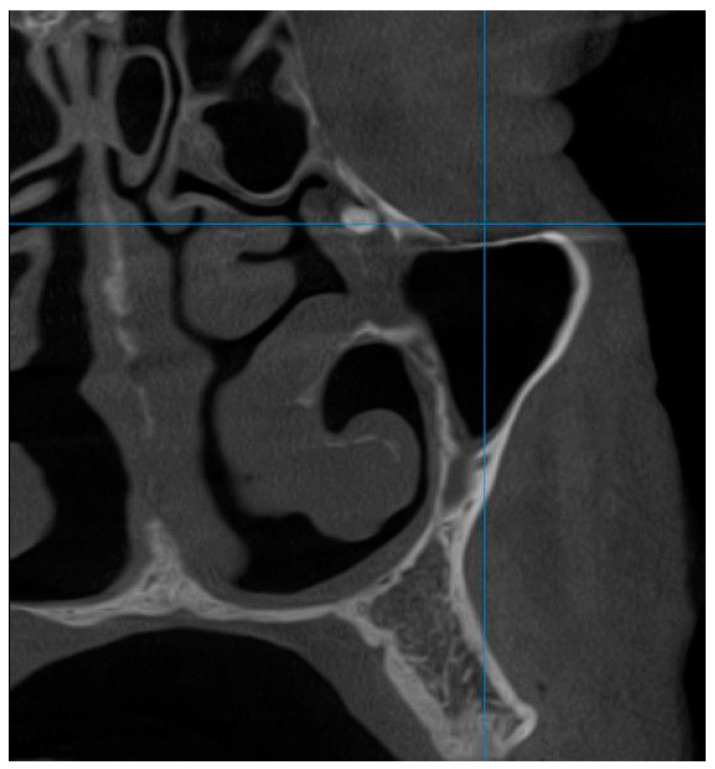
Foreign body (tooth root) migrated into the left maxillary sinus following the extraction of a wisdom molar 2.8.

Polypoid lesions were observed in 35 patients, representing 17.5% of the total examined, and their presence correlated with the patient’s age group according to the Pearson Chi-square test, which showed a test value of 11.871 with two degrees of freedom and a significance of 0.003. Sinus floor bone discontinuity was found in 43 (21.5%) of the examined patients and it was also correlated with age group, given the Pearson Chi-square test value of 16.515 with six degrees of freedom and a significance of 0.011. Sinus floor discontinuity was associated with bone graft or dental implants in 36 (83.72%) of the evaluated subjects; 5 (13.88%) of the sinus floor discontinuity cases were associated with dental extractions, and in the remaining 2.4%, the causes could not be specified. Bone thickening was found in seven subjects, i.e., in 3.5% of the cases, and this parameter was correlated with the sex of the patients, according to the results indicated by the Pearson Chi-square test, with a test value of 5.260, a degree of freedom, and a significance threshold value of *p* = 0.022. Thus, of the total number of subjects showing bone thickening on CBCT images, 85.7% were female and 14.3% were male. The presence of anthrocytes was observed in 4 subjects, representing 2%, and opacification (Figure 1) in 16 patients, representing 8%. Intrasinonasal foreign bodies (Figure 2) were found in three (1.5%) patients.

## 4. Discussion

In our study, the most common variation found in the assessed patients was the pneumatization of the maxillary sinus, which can be described by an extension of the cavity towards the alveolar ridge, the palatine, and zygomatic bone or in the direction of the orbital region. This finding is in agreement with the conclusions of other studies [1,16], which, after analyzing CBCT images, reported a frequency of more than 50% of this anatomical variation. The phenomenon of pneumatization occurs mainly due to the loss of maxillary teeth in the posterior region, which leads to atrophy of the maxillary bone and reduces considerably the thickness of the bone intended for implant insertion [1,17]. In our study, pneumatization was found in 81.5% of the evaluated subjects, and of the total reported cases, 32.5% were found in the alveolar site and 67.5% in multiple sites. Pneumatization affected 59.5% of male subjects and 40.5% of female subjects, but it was not possible to statistically correlate the frequency of the pathology with the gender of the patients. The pneumatization of the maxillary sinus was found in 33.7% of patients aged between 20 and 35 years, in 55.8% of patients aged between 36 and 60 years, and in 10.4% of patients aged between 61 and 81 years. No correlation could be found between the patient’s age and the frequency of occurrence of this anatomical variation. The treatment of this sinus cavity quantitative modification is achieved by the maxillary sinus floor elevation technique, which can be performed by the crestal or lateral fenestration approach [18].

Maxillary sinus hypoplasia is an anomaly caused by the underdevelopment of the sinus during the embryonic phase or later, as a result of trauma or iatrogenesis [1]. It is characterized by clogging of the air passages and thickening of the sinus mucosa. Ineffective sinus ventilation and obstruction of the sinus passages or sinus drainage, contraindicate sinus floor elevation treatment [18,19]. In our study, hypoplasia was found in a low percentage of subjects, 6.5% of whom 53.8% were male and 46.2% were female. The presence of hypoplastic sinuses has been associated with more frequent perforation of the sinus membrane during the sinus floor elevation treatment [18,20]. In other studies, hypoplastic mucosa has been correlated with the patient’s ethnicity [18,21]. In our study, however, no statistical correlation was found between the frequency of occurrence of this anatomic variation and the patient’s sex or age group distribution.

The antral septum and the exostoses are bone formations, the first with a pointed shape and the second with a rounded shape that can be located on any of the walls of the sinus cavity [22]. In the present study, we found the antral septum in 48.5% of the CBCT-analyzed images and the exostoses in 1.5%. Statistical analysis was unable to correlate the frequency of occurrence of these two sinus anatomic variations with the distribution of subjects by sex or age group. Previous studies show a similar frequency of occurrence of the antral septum in approximately 50% of the evaluated subjects. This aspect is responsible for the increased risk of injury of the sinus membrane during the sinus floor lift procedure, which will lead to the resorption of the bone graft or the development of acute or chronic sinusitis [23,24]. The surgical procedure of sinus floor elevation would also be hampered by the presence of antral septa [1,25].

The maxillary sinuses are lined by a respiratory-type epithelium characterized by an average thickness of about 1 mm under normal conditions and can thicken up to 10 times in acute or chronic sinus pathology. Mucosal thickness of more than 3 mm is considered pathological [16]. A study of cadavers showed that in subjects with sinus pathology, it was in the range of 0.3 to 0.8 mm [26], while another study proposed that the mucosa should be considered thickened when it exceeds 1 mm thickness [18]. The prevalence of mucosal thickening was 40.5%, which is in complete agreement with the results of other studies [21,26]. However, mucosal thickening is not necessarily a sign of a pathology but may be a consequence of allergic reactions or aggression caused by cigarette smoke. Mucosal thickening has been reported as a contraindication to the sinus floor lifting procedure [23]. In our study, we evaluated the thickness of the mucosa based on measurements using a digital ruler, and the results showed a thickness of ≤3 mm in 59.5% of subjects, and in the remaining 40.5%, the thickness was greater than 3 mm. Statistical analysis of the data showed us a positive correlation between the frequency of occurrence of mucosa with a thickness greater than 3 mm and the age group of the subjects, due to a Pearson test value of 65.605 with two degrees of freedom and a significance of <0.001. For subjects in the age group of 20–35 years, mucosal thickening of less than 3 mm was identified in 98.5% of subjects. In the age group of 61–81 years, mucosal thickening of <3 mm was observed in 88.8% of subjects. From a radiologic point of view, paranasal sinus inflammation can be detected based on typical images of sinus opacification and mucosal thickening. The phenomenon of sinus opacification is a characteristic radiologic feature of bacterial rhinosinusitis. The 8% prevalence of the opacification phenomenon was higher compared to some studies that showed a value of 4%, or lower compared to others that reported values between 12 and 35% based on CBCT images [21,26,27].

Discontinuity of the sinus floor was found in 21.5% of the subjects, 83.7% of which were the consequence of bone grafts, 11.6% of dental extractions, and 4.7% of which were due to unclear causes. The frequency of lesions could not be correlated with gender or age group. As reported in other studies, sinus floor discontinuity is a frequent consequence of surgery or infection involving the maxillary sinus, implants, bone grafts, or dental extractions [11,28,29].

Polypoid lesions, represented by the antrochoanal polyp and the mucous retention cyst [1], were also considered when evaluating anatomic variations in the maxillary sinus. Both cystic formations show similar radiologic features and are difficult to differentiate [6]. Cysts and polyps are benign tumors with a reported frequency of between 1 and 25%, so the percentage of 17.5% found in this study falls within this range [18,21,26,30]. Some authors consider the presence of benign formations in the maxillary sinus to be a clear contraindication for surgical treatment in the maxillary sinus [30]. In our study, they were found in 17.5% of the subjects, affecting males in 65.7% and females in 38.7% of the cases. The presence of polypoid lesions was found in 57.1% of subjects aged between 20 and 35 years, 42.9% of subjects aged between 36 and 60 years, and absent in subjects aged over 61 years. Polypoid lesions were correlated with the patient age group. The absence of polypoid lesions in subjects older than 61 years of age may be attributed to the time-regressive nature of these cysts [31,32,33].

The anthroliths found in a very low percentage of 2% represent pathologic formations as calcifications in the sinuses developed as a result of abnormal deposition of mineral salts [1,34]. Radiologically, they appear as an opaque mass that requires surgical removal only when it produces clinical symptoms [1,35]. The presence of foreign bodies in the maxillary sinus cavity is the consequence of oro-antral or alveolar communication or a surgical procedure [9]. In our study, foreign bodies were found in the sinus cavity in 1.5%, and their presence was not correlated with patient gender or age group.

Considering the plethora of lesions or anatomic variations detected by analyzing CBCT images, the importance of this procedure in the preoperative stage of sinus floor elevation or implant treatment can be emphasized. The CBCT technique is preferred to other radiologic methods due to the clarity of the image generated on the basis of which anatomic variations or lesions can be identified [18].

A limitation of this study is determined by the use of a relatively small sample of subjects on which it is very difficult to generalize the obtained results. Also, the subjects included in the study were patients who were recommended to undergo CBCT as part of the preoperative evaluation of implant treatment, so the results cannot be extrapolated to the general population. The results of this study should be interpreted with caution considering the study’s retrospective nature conducted in a dental radiology center.

## 5. Conclusions

The null hypothesis was supported for some anatomical variations, such as pneumatization and antral septa where no significant correlations with the patient’s age or gender were found, but it was rejected for others, like mucosal thickening, polypoid lesions, bone thickening, and sinus floor discontinuity, where significant correlations were identified.

The results of this study emphasize the importance of a thorough CBCT evaluation of the maxillary sinus in patients proposed for dental implant treatment.

## Figures and Tables

**Table 1:** The frequency gender and age group distribution of anatomical variations and lesions.

Anatomic Variation	Frequency		Gender Distribution	*p* Value ^1^	Age Group Distribution	*p* Value ^1^
Pneumatization	163 (81.5%) -alveolar 32.5% -multiple sites 67.5%		M: 97 (59.5%) F: 66 (40.5%)	3.246 ^1^ 0.53 ^2^	A: 55 (33.7%) B: 91 (55.8%) C: 17 (10.4%)	4.304 ^1^ 0.116 ^2^
Antral septa	97 (48.5%)		M: 52 (53.6%) F: 45 (46.4%)	5.641 ^1^ 0.255 ^2^	A:35 (36.1%) B: 52 (53.6%) C: 10 (10.3%)	1.462 ^1^ 0.481 ^2^
Hypoplasia	13 (6.5%)		M: 7 (53.8%) F: 6 (46.2%)	0.141 ^1^ 0.842 ^2^	A: 7 (53.8%) B: 5 (38.5%) C: 1 (7.7%)	2.498 ^1^ 0.287 ^2^
Exostosis	3 (1.5%)		M: 2 (66.6%) F: 1 (33.3%)	0.128 ^1^ 0.597 ^2^	A:1 (33.3%) B:2 (66.6%) C:0	0.304 ^1^ 0.859 ^2^
Pathological factors						
Mucosal thickening	>3 mm 81 (40.5%)		M: 40 (49.4%) F:41 (50.6%)	2.8061 0.063 ^2^	A: 1 B: 71 C: 9	65.605 ^1^ <0.001 ^2^
	≤3 mm 119 (59.5%)		M:73 (61.3%) F:46 (38.7%)		A: 67 B: 44 C: 8	
Polypoid lesion	35 (17.5%)		M: 23 (65.7%) F: 12 (34.3%)	1.466 ^1^ 0.153 ^2^	A: 20 (57.1%) B: 15 (42.9%) C: 0	11.871 ^1^ <0.001 ^2^
Bone thickening	7 (3.5%)		M: 1 (14.3%) F: 6 (85.7%)	5.260 ^1^ 0.022 ^2^	A: 2 (28.6%) B: 4 (57.1%) C: 1 14.3%)	0.349 ^1^ 0.840 ^2^
Antrolith	4 (2%)		M: 3 (75%) F: 1 (25%)	0.568 ^1^ 0.414 ^2^	A: 1 (25% B: 2 (50%) C: 1 (25%)	1.444 ^1^ 0.486 ^2^
Sinusal floor discontinuity	43 (21.5%)	Bone graft 36 (83.7%)	M: 20 (55.5%) F: 16 (44.4%)	2.679 ^1^ 0.444 ^2^	A: 18 (50%) B: 11 (30.6%) C: 7 (19.4%)	16.515 ^1^ 0.011 ^2^
		Extraction 5 (11.6%)	M: 3 (60%) F: 2 (40%)		A: 2 (40%) B: 3 (60%) C: 0	
		Unclear reason 2 (4.7%)	M: 0 F: 2 (100%)		A: 0 B: 2 (100% C: 0	
Foreign body	3 (1.5%)		M: 1 (33.3%) F: 2 (66.6%)	0.665 ^1^ 0.403 ^2^	A: 0 B: 2 (66.6%) C: 1 (33.3%)	3.290 ^1^ 0.193 ^2^
Opacifiation	16 (8%)		M: 7 (43.8%) F: 9 (56.2%)	1.150 ^1^ 0.208 ^2^	A: 6 (37.5%) B: 9 (56.3%) C: 1 (6.2%)	0.171 ^1^ 0.918 ^2^

^1^ Pearson Chi-square test value. ^2^ Significance *p* value. M: male; F: female; A: age group 20–35 years; B: age group 36–60 years; C: age group 61–81.

## Data Availability

The original contributions presented in the study are included in the article, further inquiries can be directed to the corresponding author.
